# Core Outcome Set for GROwth restriction: deVeloping Endpoints (COSGROVE)

**DOI:** 10.1186/s13063-018-2819-9

**Published:** 2018-08-22

**Authors:** Patricia Healy, Sanne Gordijn, Wessel Ganzevoort, Irene Beune, Ahmet Baschat, Asma Khalil, Louise Kenny, Frank Bloomfield, Mandy Daly, Aris Papageorghiou, Declan Devane

**Affiliations:** 1 Health Research Board – Trials Methodology Research Network, Galway, Ireland; 2 School of Nursing and Midwifery, National University of Ireland Galway, Galway, Ireland; 3 Department of Obstetrics and Gynecology, University Medical Center Groningen, University of Groningen, Groningen, The Netherlands; 4 Department of Obstetrics and Gynecology, Academic Medical Center, University of Amsterdam, Amsterdam, The Netherlands; 5 Johns Hopkins Center for Fetal Therapy, Baltimore, USA; 6 Fetal Medicine Unit, St George’s University and St George’s University Hospitals NHS Foundation Trust, London, UK; 7 The Irish Centre for Fetal and Neonatal Translational Research (INFANT), University College Cork, Cork, Ireland; 8 Liggins Institute, University of Auckland, Auckland, New Zealand; 9 Advocacy and Policymaking, Irish Neonatal Health Alliance, Wicklow, Ireland; 10 Nuffield Department of Obstetrics and Gynaecology, Oxford Maternal and Perinatal Health Institute (OMPHI), University of Oxford, Oxford, UK

**Keywords:** Core outcome sets, Delphi, Methodology, Foetal growth restriction, Pregnancy

## Abstract

**Background:**

Foetal growth restriction (FGR) refers to a foetus that does not reach its genetically predetermined growth potential. It is well recognised that growth-restricted foetuses are at increased risk of stillbirth, foetal compromise, early neonatal death and neonatal morbidity. Later in life, they are prone to health problems, including increased risk of cardiovascular diseases and neurodevelopmental disorders. Interventions for preventing and treating FGR have been studied in many trials, but evidence is often difficult to synthesise and compare because of differences in the selection and definition of outcomes. To enable future trials to measure similar, meaningful outcomes, we are developing two core outcome sets (COS) – one for prevention and the other for treatment of FGR.

**Methods:**

We will review the literature to identify previously reported outcomes. An international panel of relevant stakeholders who have experience of FGR (parent or carer of a baby that was growth restricted, health professional involved in the care of mothers and babies affected by FGR, a person with expertise in FGR research) will rate the importance of each of those outcomes in a series of three sequential online rounds of a Delphi study. Participants will be able to add items to the proposed list in round 1. A final face-to-face consensus meeting will be held with representatives of each stakeholder group at which a final list of outcomes for inclusion in the COS will be agreed.

**Discussion:**

The development of COSs in FGR will ensure the collection and reporting of a minimum dataset agreed by stakeholder consensus and will reduce inconsistencies in the reporting of outcomes across relevant trials. Such standardisation in the reporting of outcomes will improve synthesis of evidence and generalisability of knowledge in the future by reducing heterogeneity in outcomes between trials and thus improve the results of systematic reviews and meta-analyses. Ultimately, we hope that the COSs will lead to an improvement in the quality of evidence-based clinical practice, enhance patient care, and improve the quality and consistency of research.

**Trial registration:**

Not applicable. This study is registered in the Core Outcome Measures for Effectiveness (COMET) database.

## Background

Several factors adversely influence foetal growth, including maternal disease, problems with establishment of the utero-placental circulation in early pregnancy, maternal nutritional deprivation, chromosomal and other abnormalities in the foetus and/or placenta, pregnancy-related medical conditions such as gestational diabetes and pre-eclampsia, and exposure to adverse environmental conditions such as high altitude, toxins including smoking, drugs and other teratogens, infections and foetal metabolic diseases [1, 2]. It is well recognised that foetuses with foetal growth restriction (FGR) are at increased risk of stillbirth, foetal compromise, early neonatal death and neonatal morbidity [3, 4]. Evidence is increasing that exposure to an adverse foetal nutritional environment also causes lifelong changes in the risk profile for metabolic disease, neurodevelopment and cardiac disease [5, 6], also observed in gene expression, with increased risk of later metabolic diseases seen in neonates born both small- and large-for-gestational-age [7].

Although the heterogeneity of pathologies leading to FGR results in a wide range of perinatal prognoses, outcomes differ between the underlying causes. Ultrasound identification of foetuses with abnormal growth is often based on a single size parameter, such as abdominal circumference, or a single (but composite) measure of estimated foetal weight [8]. Small-for-gestational-age babies are usually defined as estimated foetal weight or abdominal circumference below the 10th percentile on a foetal size chart. Nevertheless, current tests do not distinguish well between foetuses at risk of death due to a pathologic process (i.e. the growth restricted foetuses) from foetuses that are small but are otherwise likely to experience good perinatal outcomes without medical intervention. Single size parameters also fail to detect foetuses experiencing FGR later in pregnancy that may still be in the ‘healthy’ range (for example, foetuses that were destined to be on the 50th centile for size but are actually on the 15th) thus not small-for-gestational-age but nevertheless still FGR.

Doppler and other functional studies have proven useful in identifying small foetuses at preterm gestations at risk of demise [9]; however, the utility of these tests at later gestation (after 32 weeks) is more limited [10]. Recently, a consensus definition for both early and late FGR was developed that incorporated biometrical as well as functional parameters in which foetuses with measurements above the 10th percentile may be diagnosed as FGR [11].

Antenatal care has foetal growth monitoring as a central aim in order to identify pregnancies with poor growth that may benefit from surveillance of foetal well-being; this is based on the premise that timely intervention in response to evolving foetal compromise can improve outcomes. Screening for growth restriction in pregnancy may target detection of pregnancies at risk of poor growth or later in pregnancy when growth restriction has occurred, with the aim of preventing stillbirth. Effective preventative interventions in women at risk of growth restriction detected early in pregnancy are few, with aspirin currently recommended for clinical use in a subgroup of women at high risk of pre-eclampsia or FGR due to failure of spiral artery remodelling (placental insufficiency due to maternal vascular malperfusion) prior to 16 weeks’ gestation [12] and women who smoke being supported to cease [13]. Later in pregnancy, antenatal corticosteroids and planned early delivery has the potential to avert stillbirth and prematurity-related risks, although moderate to late preterm babies are still at risk of neonatal and longer-term complications [14, 15].

Clinically important outcomes potentially relevant to FGR are diverse and include both maternal and foetal outcomes. However, there is heterogeneity in the outcomes measured and reported in studies evaluating the effects of interventions for the prevention and treatment of FGR [16–18]. Such heterogeneity limits the ability of clinicians, researchers and reviewers to compare, contrast and synthesise information on similar interventions for similar populations across studies. One of the suggested ways to address this is to develop and apply agreed standardised sets of outcomes, known as ‘core outcome sets’ (COS). A COS is a minimum set of agreed, standardised outcomes to be measured and reported worldwide in all trials on a specific condition [19]. This minimises the substantial heterogeneity in choice of outcome measures, allowing the efficient comparison and synthesis of trials [20]. and encourages a more complete reporting of outcomes, which limits reporting bias [21].

## Objective

To develop COSs for prevention and management of FGR.

## Methods

The design of this COS project will be guided by The Core Outcome Set-STAndards for Development (COS-STAD). A step-wise refinement approach will be used, consisting of four discrete, yet complementary, sub-work packages (sWP), whereby each sWP will inform the next sWP.

- sWP1: A comprehensive review to identify reported outcomes in FGR studies
- sWP2: Development, through an online Delphi consensus process, of two preliminary COSs for interventions for the (1) prevention and (2) treatment of FGR.
- sWP3A: Face-to-face consensus meeting to discuss and agree on the final FGR COSs;
- sWP4: Development of a dissemination and implementation strategy for the final (1) prevention and (2) treatment of FGR COSs

### sWP1: A comprehensive review to identify reported outcome measures in clinical trials in FGR

#### Objective

To conduct a review of all the mentioned outcomes in FGR studies in 1994, 2004 and 2014 to identify what has been reported. The conduct of the review will adhere to standard searching and selection strategies.

#### Inclusion criteria

- Participants: pregnant women with or at risk of having a growth restricted foetus or growth restricted newborn.
- Any study that investigated risk factors, diagnosing, prevention, treatment, follow-up or any other aspect of FGR. We will exclude case reports, studies that do not concern FGR, letters to the editors, commentaries, expert opinion reviews and animal studies.
- All outcomes reported in the included FGR studies will be collected.

#### Search methods for identification of studies

A comprehensive search for published literature on FGR will be conducted in the Cochrane Central Database, PubMed and MEDLINE/EMBASE electronic databases. The combinations of keywords and terms that will be used in the search are Major MeSH and MeSH on foetal or intrauterine growth restriction or retardation and small for gestational age in the title. Our final search is: ((fet* OR foet*) OR (intra-ut* OR intraut*)) AND ((grow* AND (rest* OR retard*)) OR (SGA OR small for gestational age OR FGR OR IUGR) with filters for Language (English) and Search field (title). Articles from Jan 1 until December 31, 1994, from Jan 1 until December 31, 2004, and from Jan 1 until December 31, 2014, will be selected.

#### Data collection and management

A purposively developed data abstraction form will be used to collect the relevant data, i.e. the key characteristics of the eligible studies and the reported outcomes. The identified records will be assessed by two reviewers according to the criteria above. Disagreement between reviewers on abstracted data will be resolved by reading the full text or, if necessary, by group discussion. The reported outcomes will include both those that are mentioned as being measured in the methods section of the relevant study report or record, and those for which findings are presented. Outcomes will be categorised per type of intervention under evaluation (i.e. screening, prevention or treatment intervention), and whether they are primary or secondary. Where studies focus on more than one intervention type, outcomes will be extracted according to type of intervention that the outcome is being reported for. Where this is not clear from the study, the outcome will be extracted into a separate column titled ‘uncertain’.

#### Data analysis

Once data from all included studies have been extracted, a list of unique outcomes of FGR will be developed. These outcomes will be categorised in domains, e.g. maternal outcomes, neonatal outcomes or long-term outcomes, with subcategories as appropriate. A unique list of outcomes that are common to both treatment and prevention will be formed during the analysis phase, and a separate list will be made for any outcome extracted into the ‘uncertain’ column during the data extraction process. These final lists of outcomes will inform sWP2.

### sWP2: Development of a preliminary COS for use in the (1) prevention and (2) treatment of FGR

#### Objective

Using the outcomes identified by sWP1, an online, electronic Delphi method will be used for developing the preliminary FGR COSs in sWP2.

#### Delphi procedure

The Delphi method facilitates a means of consensus building by using a series of sequential questionnaires to collect data from a panel of experts, service users and other stakeholders on the topic under investigation, and has been used previously in COS development [22]. Originally developed by the RAND Corporation to come to consensus on warfare technology in the 1950s, the Delphi process is iterative and based on the scoring of a series of structured statements that are revised, fed back to the participants and repeated in multiple rounds, in increasing detail, until consensus has been reached [23].

#### Recruitment of Delphi participants

The target population reflects the perspectives of a variety of stakeholders in FGR studies. Purposeful sampling to approach people with informed opinions or known expertise in FGR will be used. Those potential participants will be asked to forward the invitation to others whom they regard as having the required expertise, thereby facilitating snowball sampling. We will invite women and their partners as users of maternity services, midwives, obstetricians, paediatricians/neonatologists, family doctors, policymakers and groups with specific expertise/interest in FGR care and perinatal research. The target sample will be accessed through mass invitational emails, electronic discussion lists and professional organisations. For example,

- Colleges of Obstetricians and Gynaecologists (UK, Australia, Canada, Netherlands, Germany)
- College of Physicians
- Cochrane Pregnancy & Childbirth Reviewers’ Group
- Cochrane Pregnancy & Childbirth consumer networks
- European Perinatal Epidemiology Network
- Association for Improvements in Maternity Services (Ireland and UK)
- European Network of Childbirth Associations
- European Midwives Association
- The International Society of Ultrasound in Obstetrics & Gynecology
- Perinatal societies
- Patient groups such as SANDS, INHA, MUMSnet, the Dutch HELLP foundation and VOC (parents of incubator children)

Note that this list is not exhaustive; rather, it merely provides some examples of e-discussion lists that might be accessed.

We will also invite those who have participated in previous work in this area and other experts in FGR who have published on FGR as identified in sWP1. We will ask all potential participants to suggest colleagues who should be in the Delphi panel, aiming for international coverage of the Delphi panel.

Recruitment of users of maternity services will be via the electronic discussion e-mail list manager. The manager (or chairperson of the group, details of which are publicly available on the websites of the groups listed above) will be emailed with information on the survey and a request to distribute an invitation email to members on their email lists. The list managers will have an opportunity to contact the researchers directly to clarify any issues or to seek further information about the survey and the research prior to deciding. The distribution of the survey, ultimately, will be at the discretion of the email list manager. There is precedent for survey distribution on matters related to maternity care by these groups (for example, http://www.irishhealth.com/article.html?id=23473). The recruitment of users of maternity services will be guided by a maternity service user who sits on our working group and acts as our patient and public involvement research partner.

An invitation e-mail will be circulated to potential and suitable Delphi participants. We will provide every potential participant with an explanatory email and a video in which the need for the study, as well as what participation involves and why it is important, will be explained. We will explain the principles of a COS as a minimum set of outcomes that are mandatory to report in future studies (the difference between ‘core’ outcome and ‘desired’ outcomes). Individuals who wish to participate will be asked to click on a link that will enable them to register with the DelphiManager software system, which we are using for this study. On receipt of this, the researcher will forward further information, instructions and the round 1 survey instrument, which is accessible only after the potential participant has indicated their formal consent. We will ask all respondents to forward the invitation email to other potential participants who might be interested in the study.

#### Data collection and analysis

An online Delphi with a series of three sequential rounds is planned. Questionnaires will be completed using DelphiManager, which is a web-based system designed to facilitate the building and management of Delphi surveys. In each round, panel members will receive an e-mail with a unique link to the questionnaire. Responses to each round will be collated, analysed, and redistributed to participants with their individual response to each item for further comment in successive rounds. Each participant will also be shown the proportion of participants within each stakeholder group that rated the outcome at each of the 1–9 points on the rating scale.

Each round will have a response closing date 21 days after the date of distribution of the survey. Responses will be monitored and an e-mail reminder will be sent to anyone who has not responded by day 14, with a final reminder after 19 days. The number of participants completing each round and attrition across rounds will be documented.

Within the Delphi process, ratings of all panel members are weighed equally within their own stakeholder category. Panel members who did not enter a round will not be invited for subsequent rounds.

#### First round

The first-round instrument will contain the rating instrument with outcomes identified in the review (sWP1) and a short questionnaire seeking participant demographic data. Each outcome will have an associated plain English definition. We will group our outcomes under relevant domains such as maternal, foetal, neonatal, physiological, mortality, functioning, etc. To ensure completeness of outcomes, participants in this round will also be invited to add up to two further ‘new’ outcomes that they would consider important or relevant for inclusion in either the prevention or treatment of FGR COSs.

The panel will be asked to rate the outcomes for FGR prevention and treatment on a 9-point Likert-scale. Typically, 1–3 signifies an outcome is of limited importance, 4–6 important but not critical, and 7–9 critical [22]. We will also include an ‘unable to score’ category to allow an option for participants who may feel that they do not have the level of expertise to score certain outcomes.

#### Second round

The second-round instrument will contain all outcomes from round 1. Only those participants who have completed the first round will be invited to participate in round 2. All additional outcomes suggested by at least two participants and not already included in round 1 will be included in round 2. For each outcome from round 1, the rating results (i.e. the proportion of participants rating each point on the 9-point rating scale) from each stakeholder group (maternity service users, midwives, obstetricians, paediatricians/neonatologists, and groups with expertise in FGR and research) will be presented numerically in the form of proportions within DelphiManager. Proportions will be produced for each stakeholder group separately. In addition, each participant will be able to see their individual round 1 score, which they may compare against the distribution of responses for their respective stakeholder group and the other stakeholder groups. Participants will be asked to re-rate the importance of each outcome with knowledge of their and other group’s previous ratings. In addition, participants will be asked to rate the newly identified outcomes from round 1. All ratings will use the same Likert-type scale that was used in round 1. The round 2 Delphi instrument will contain a question asking the participant about their willingness and availability to attend the subsequent face-to-face consensus meeting.

#### Third round

In round 3, participants who completed round 2 will be presented with the outcomes from round 2 that were rated as critical (7–9 on Likert scale) for inclusion in the COSs by at least 70% of all respondents and those rated as of limited importance (1–3 on Likert scale) by 15% or less of all respondents. Each retained outcome will be presented as in round 1. Participants will be asked to re-rate the retained outcomes from round 2 using the same Likert-type scale that was used in rounds 1 and 2. All outcomes judged ‘consensus in’ and those judged neither ‘consensus in’ nor ‘consensus out’ (Table 1) will be taken forward to the face-to-face consensus meeting.

**Table 1:** Consensus classification

Consensus classification	Description	Definition
Consensus in	Consensus that outcome should be included in the core outcome set	70% or more participants scoring as 7 to 9 AND < 15% participants scoring as 1 to 3
Consensus out	Consensus that outcome should not be included in the core outcome set	70% or more participants scoring as 1 to 3 AND < 15% of participants scoring as 7 to 9
Neither consensus in nor out (no consensus)	Uncertainty about importance of outcome so retain for next round	Anything else

### sWP3: Consensus meeting

#### Objective

Consensus on the final (1) prevention and (2) treatment of FGR COSs will be achieved through a face-to-face meeting with representatives of key stakeholders in FGR clinical care and research.

#### Participants

Participants for the meeting will be selected from Delphi completers who indicated that they were interested in attending. The consensus group will include, at a minimum, three of all stakeholder groups (maternity service users, midwives, obstetricians, paediatricians/neonatologists, and groups with expertise in FGR and research).

#### Schedule

A full-day meeting is proposed. Materials will be distributed in advance of the meeting date with the intention that participants may consider these individually, but not with others within or outside of their stakeholder group (i.e. participants will be actively discouraged from discussing these materials with others in advance of the meeting). To achieve effective consensus, the facilitator will ensure that the meeting is (1) collaborative, (2) cooperative and non-competitive, and (3) inclusive with equal input from all participants. The purpose of the meeting will be discussed, i.e. to finalise the core outcomes for each of the COSs. All outcomes judged ‘consensus out’ in the Delphi rounds will be presented as a set. The group will be asked if they support the removal of these outcomes. Next, all outcomes judged ‘consensus in’ will be presented individually and the group asked, following discussion, to vote whether the outcome should be included in the respective COS. Finally, all outcomes that are judged neither ‘consensus in’ nor ‘consensus out’ will be discussed individually and the group will vote whether the outcome should be included.

### sWP4 dissemination and implementation strategy

Developing and agreeing on a COS for use in studies on a specific condition provides for effective outcome measurement in studies, and for synthesis of evidence in systematic reviews, and should reduce waste in research [22, 24]. However, for a COS to be truly beneficial, it must be used in all studies on a given condition, where appropriate. For this to occur, wide dissemination of the developed COS is required. COS developers are recommended to consider engagement with the relevant Cochrane Review Groups, clinical guideline developers, research funders, journal editors, regulators such as research ethics committees, and trial registries. The CROWN initiative, led by journal editors, in resolving to harmonise outcome reporting in women’s health research, supports the implementation of COSs. This sWP will develop a detailed dissemination and implementation strategy to ensure effective dissemination of the final FGR COSs, to raise awareness of the developed COSs and to encourage their use in future studies in FGR.

## Discussion

Although there is an extensive list of planed/ongoing and completed COSs in the ‘pregnancy and childbirth’ health area on the Core Outcome Measures for Effectiveness in Trials (COMET) website (www.comet-initiative.org/studies/search), there is currently no published COS for FGR. We acknowledge the potential for overlap between some outcomes in this COS and other COSs such as preterm birth, stillbirth and pre-eclampsia, but believe that a separate COS for FGR, and for other conditions, is valuable and necessary. We propose the development of two COSs (prevention and treatment) to be measured in future studies on pregnancies complicated by FGR. The development of COSs in FGR will ensure the collection and reporting of a minimum dataset agreed by stakeholder consensus and will reduce inconsistencies in the reporting of outcomes across relevant trials. Such standardisation in the reporting of outcomes will improve the synthesis of evidence and generalisability of knowledge in the future by reducing heterogeneity between trials and thus improve the results of systematic reviews and meta-analyses.

The methodology we have chosen (selection of participants, Delphi rounds, consensus meeting) is based on methods successfully used in previously developed COSs. The COS process using the Delphi method is a widely accepted methodology. However, it must be acknowledged that the methods used lack a robust evidence base for all components. According to Gargon et al. ([25], p. 141), the concept of a COS is still being established, and little is known about what should inform developers’ methodological choices. The credibility of a COS depends on the use of sound methodology; we will consider the potential impact of our methodological decisions in our final study report.

This COS will identify outcomes to be measured (‘what’ to measure). We acknowledge that further work will be needed to identify the tools, timing, etc. required to measure outcomes in the COS (‘how’ to measure). We acknowledge that it will be necessary for us to limit our search to English papers as we do not have the resources for translating non-English papers and recognise this as a potential limitation.

Inviting relevant stakeholders who have experience of FGR (for example, parent or carer of a baby that was growth restricted, health professional involved in the care of mothers and babies affected by FGR, a person with expertise in FGR research) to take part in this study will ensure that the resulting COS has broad consensus, is relevant and is wide reaching.

Ultimately, we hope that the standardisation in trial outcomes will lead to an improvement in the quality of evidence-based clinical practice, enhance patient care and improve the quality and consistency of research.

### Project status

The review of the literature is complete and the list of outcomes for the round 1 Delphi has been compiled. We are currently preparing to recruit participants to the Delphi study. The final COS is expected by August 2018.

## Abbreviations

COS: core outcome set
FGR: foetal growth restriction
sWP: sub-work packages

## References

[1] Mayer C, Joseph K. Fetal growth: a review of terms, concepts and issues relevant to obstetrics. Ultrasound Obstet Gynecol 2013; 41:136–45. 10.1002/uog.11204 22648955

[2] Dall’Asta A, Brunelli V, Prefumo F, Frusca T, Lees C (2017). Early onset fetal growth restriction. Matern Health Neonatol Perinatol.

[3] Serena C, Marchetti G, Rambaldi M (2013). Stillbirth and fetal growth restriction. J Matern Fetal Neonatal Med.

[4] Unterscheider J, O’Donoghue K, Daly S, Geary MP, Kennelly MM, McAuliffe FM, Hunter A, Morrison JJ, Burke G, Dicker P, Tully E, Malone FD (2014). Fetal growth restriction and the risk of perinatal mortality–case studies from the multicentre PORTO study. BMC Pregnancy Childbirth.

[5] Gluckman PD, Hanson MA, Cooper C, Thornburg KL (2008). Effect of in utero and early-life conditions on adult health and disease. N Engl J Med.

[6] Harding JE (2001). The nutritional basis of the fetal origins of adult disease. Int J Epidemiol.

[7] Lillycrop KA, Burdge GC (2011). The effect of nutrition during early life on the epigenetic regulation of transcription and implications for human diseases. J Nutrigenet Nutrigenom.

[8] RCOG. Small-for-gestational-age Fetus, Investigation and Management. Green-top Guideline No. 31. 2013. www.rcog.org.uk/en/guidelines-research-services/guidelines/gtg31/. Accessed 16 July 2018.

[9] Alfirevic Z, Stampalija T, Gyte GM (2010). Fetal and umbilical Doppler ultrasound in high-risk pregnancies. Cochrane Database Syst Rev.

[10] Oros D, Figueras F, Cruz-Martinez R, Meler E, Munmany M, Gratacos E (2011). Longitudinal changes in uterine, umbilical and fetal cerebral Doppler indices in late-onset small-for-gestational age fetuses. Ultrasound Obstet Gynecol.

[11] Gordijn S, Beune I, Thilaganathan B, Papageorghiou A, Baschat A, Baker P, Silver R, Wynia K, Ganzevoort W (2016). Consensus definition of fetal growth restriction: a Delphi procedure. Ultrasound Obstet Gynecol.

[12] Askie LM, Duley L, Henderson-Smart DJ, Stewart LA, PARIS Collaborative Group (2007). Antiplatelet agents for prevention of pre-eclampsia: a meta-analysis of individual patient data. Lancet.

[13] McCowan LM, Dekker GA, Chan E, Stewart A, Chappell LC, Hunter M (2009). Spontaneous preterm birth and small for gestational age infants in women who stop smoking early in pregnancy: prospective cohort study. BMJ.

[14] Brown HK, Speechley KN, Macnab J, Natale R, Campbell MK (2014). Neonatal morbidity associated with late preterm and early term birth: the roles of gestational age and biological determinants of preterm birth. Int J Epidemiol.

[15] Crump C, Sundquist K, Sundquist J, Winkleby MA (2011). Gestational age at birth and mortality in young adulthood. JAMA.

[16] GRIT Study Group (2003). A randomised trial of timed delivery for the compromised preterm fetus: short term outcomes and Bayesian interpretation. Br J Obstet Gynaecol.

[17] Unterscheider J, Daly S, Geary MP, Kennelly MM, McAuliffe FM, O’Donoghue K, Hunter A, Morrison JJ, Burke G, Dicker P, Tully E, Malone FD (2013). Optimizing the definition of intrauterine growth restriction: the multicenter prospective PORTO Study. Am J Obstet Gynecol.

[18] Lees CC, Marlow N, van Wassenaer-Leemhuis A, for the TRUFFLE study group (2015). 2 year neurodevelopmental and intermediate perinatal outcomes in infants with very preterm fetal growth restriction (TRUFFLE): a randomised trial. Lancet.

[19] Williamson PR, Altman DG, Blazeby JM, Clarke M, Devane D, Gargon E, Tugwell P (2012). Developing core outcome sets for clinical trials: issues to consider. Trials.

[20] Devane D, Begley CM, Clarke M, Horey D, O'Boyle C (2007). Evaluating maternity care: a core set of outcome measures. Birth.

[21] Clarke M (2007). Standardising outcomes for clinical trials and systematic reviews. Trials.

[22] Williamson PR (2017). The COMET Handbook: version 1.0. Trials.

[23] Hsu C, Sandford B. The Delphi Technique: making sense of consensus. Pract Assess Res Eval. 2007; 12(10). http://pareonline.net/getvn.asp?v=12&n=10. Accessed 12 Aug 2018.

[24] Chan AW (2014). Increasing value and reducing waste: addressing inaccessible research. Lancet.

[25] Gargon E, Williamson P, Young B (2017). Improving core outcome set development: qualitative interviews with developers provided pointers to inform guidance. J Clin Epidemiol.

